# Regulation of AKT gene expression by cisplatin

**DOI:** 10.3892/ol.2013.1132

**Published:** 2013-01-14

**Authors:** JUN ZHANG, LING-LI ZHANG, LEI SHEN, XI-MING XU, HONG-GANG YU

**Affiliations:** 1 Department of Gastroenterology, Renmin Hospital of Wuhan University, Wuhan 430060;; 2 Department of Oncology, Renmin Hospital of Wuhan University, Wuhan 430060, P.R. China

**Keywords:** AKT, reactive oxygen species, chemoresistance, colon cancer

## Abstract

The use of chemotherapy drugs for the treatment of cancer is an effective therapeutic measure. However, chemoresistance affects the effectiveness of the treatment. AKT overexpression has been observed in chemoresistance. AKT expression in colon cells induced cisplatin resistance. The present study demonstrated the role of reactive oxygen species (ROS) in the induction of AKT regulation by cisplatin through the activation of JAK2/STAT3 at the transcriptional level in colon cancer cells. HCT-116 cells treated with cisplatin exhibited increased JAK2 and STAT3 activities. Reducing the expression of JAK2 in colon cancer cells using small interfering RNA (siRNA) decreased AKT expression. The present study demonstrated that AKT activation is closely associated with chemoresistance in human tumors. The inhibition of ROS decreased the levels of AKT in colon cancer cell lines. The JAK2/STAT3 pathway was also shown to mediate AKT expression and represents a potential target for overcoming cisplatin resistance in human tumors.

## Introduction

Colorectal cancer remains one of the most prevalent health problems worldwide and it is the third most common type of cancer and the second most common cause of cancer-related mortality (1). The use of cytotoxic agents, such as cisplatin (CDDP), is an effective and important treatment for colon cancer (2,3). However, the efficacy of these drugs is limited by the effects of chemoresistance, which is associated with AKT overexpression (4). Thus, methods to decrease the expression of AKT have received much attention.

AKT/PKB is a serine/threonine kinase and belongs to a family of proteins which includes AKT1, AKT2 and AKT3. The AKT pathway regulates diverse cellular processes, including cell proliferation, differentiation, apoptosis and tumorigenesis (5,6). In addition, hyperactivation of AKT has been detected in colon cancers that have acquired CDDP resistance (7). The activation of AKT induces cell survival, while the inhibition of AKT activity increases the rate of apoptosis in numerous types of cancer cells (8). The activation of AKT has been detected in human tumors with acquired chemoresistance (9–11). These observations highlight AKT as an emerging target for overcoming chemoresistance in colon cancer.

Cytoplasmic Janus protein tyrosine kinases (JAKs) regulate multiple signaling pathways that govern cell proliferation, differentiation and apoptosis (12). The JAK kinases regulate members of the signal transducers and activators of transcription (STAT) family (13). Once STAT is tyrosine-phosphorylated by JAKs, it dimerizes and translocates to the nucleus to activate the expression of genes, such as AKT. The JAK2-STAT3 signaling pathway is activated by reactive oxygen species (ROS) and this pathway activation is inhibited by antioxidants. CDDP generates ROS and may activate the JAK2/STAT3 pathway (14). Upon phosphorylation of the tyrosine residues by JAKs, STAT3 is activated to upregulate AKT expression. The AKT pathway has been extensively investigated. However, the transcriptional regulation of AKT remains largely unknown. In the present study, a sequence from the human AKT promoter, which may contain the binding sites for STAT3, was selected. AKT is upregulated by STAT3 at the transcriptional level and the overexpression of AKT is diminished by ROS inhibition. The study demonstrated that AKT activation was closely associated with chemoresistance in human tumors. The present results also showed that the JAK2/STAT3 pathway mediates AKT expression, which represents a novel target for overcoming CDDP resistance in human tumors.

## Material and methods

### Cell culture and reagents

HCT-116 colon cancer cells were obtained from the Cell Bank of the Chinese Academy of Sciences (Shanghai, China), supplemented with 10% fetal bovine serum (FBS), 100 mg/l penicillin and 100 mg/l streptomycin and maintained at 37°C in Dulbecco’s modified Eagle’s medium (DMEM) in a humidified atmosphere of 5% CO_2_. The following reagents were used: anti-AKT, anti-STAT3, anti-phosphoJAK2 and anti-phosphoSTAT3, anti-phosphoAKT (Millipore, Billerica, MA, USA), anti-JAK2 (Cell Signaling Technology, Inc., Danvers, MA, USA), JAK2 small interfering (si)RNA, control siRNA (Santa Cruz Biotechnology, Inc., Santa Cruz, CA, USA), dichlorodihydrofluorescein diacetate (DCFDA) and *N*-acetylcysteine (NAC), (Sigma, St. Louis, MO, USA).

### Measurement of ROS production by flow cytometry

HCT-116 cells were treated with CDDP for 6 h. After washing with PBS, the cells were incubated with 33 mg/ml DCFDA (Sigma) in PBS for 30 min at 37°C. The excess probe was washed off with PBS and the labeled cells were measured using flow cytometric analysis.

### JAK2 siRNA transfection

Briefly, colon cells were transfected with JAK2 siRNA and control siRNA according to the manufacturer’s instructions. Fresh medium without antibiotics was added after 8 h of incubation and after an additional 48 h, the medium was replaced with 10% fetal bovine serum (FBS), 100 mg/l penicillin and 100 mg/l streptomycin and cell growth was maintained.

### Isolation of RNA and quantitative RT-PCR

The mRNA levels of AKT in the various CDDP-treated cells were analyzed by quantitative RT-PCR. Total RNA was extracted using TRIzol (Invitrogen, Carlsbad, CA, USA), according to the manufacturer’s instructions. The total cDNA was then used in the PCR to measure the mRNA levels of AKT. The mRNA level of actin was used as the internal control. The conditions were as follows: pre-denaturing at 94°C for 4 min and 30 cycles of 94°C for 30 sec, 56°C for 30 sec and 72°C for 25 sec. The sequence of the upstream primer used for AKT was 5′-TCT ATG GCG CTG AGA TTG TG-3′ and the downstream primer sequence was 5′-CTT AAT GTG CCC GTC CTT GT-3′. For actin, the upstream primer was 5′-CAC GAT GGA GGG GCC GGA CTC ATC-3′ and the downstream primer was 5′-TAA AGA CCT CTA TGC CAA CAC AGT-3′.

### Western blotting

The cells were solubilized in ice-cold lysis buffer (1X PBS, 1% IGEPAL CA-630, 0.5% sodium deoxycholate, 0.1% SDS and 10 mg/ml phenyl). Following 30 min of centrifugation at 13,000 × g, 4°C, the supernatants were transferred to new microcentrifuge tubes and the protein concentration of the supernatant was measured using the BCA protein assay (Pierce Biotechnology, Inc., Rockford, IL, USA) and subsequently stored at −80°C. Cell lysate (50 *μ*g) was separated on an SDS-polyacrylamide gel. Following SDS-PAGE, the proteins were transferred to nitrocellulose membranes. To detect the proteins, the membranes were blocked using 10% non-fat dry milk in Tris buffer containing 0.1% Tween-20 (TBS-T) and then incubated at 4°C overnight with anti-AKT and anti-phosphoJAK2, anti-phosphoSTAT3, anti-phosphoAKT antibodies, which were diluted in TBS-T containing 5% non-fat dry milk.

### Chromatin immunoprecipitation (ChIP) assay

The ChIP assay was performed as described previously (15). Solubilized chromatin was prepared from a total of 1×10^7^ HCT-116 cells treated with CDDP and NAC (an inhibitor of ROS). The chromatin solution was diluted 10-fold with ChIP dilution buffer (SDS lysis buffer and protease inhibitor cocktail), then added to Protein G Agarose and rotated at 4°C for 1 h. The pre-cleared chromatin solution was divided and utilized in the immunoprecipitation assays with either an anti-Stat3 antibody or an anti-actin antibody. After washing with buffers (low/high salt immune complex, TE buffer), the antibody-protein-DNA complex was eluted from the beads by resuspending the pellets in 20% SDS, 1 M NaHCO_3_ and H_2_O at room temperature for 15 min. After cross-linking, the protein and RNA were removed by incubating the sample with 1 *μ*l RNase A at 37°C for 30 min and then cross-linking with the pellets in 0.5 M EDTA, 8 *μ*l 1 M Tris-Hcl and 1 *μ*l proteinase K at 45°C for 2 h. Purified DNA was subjected to PCR with primers specific to the putative Stat3-binding site within the AKT promoter. The conditions were as follows: pre-denaturing at 94°C for 4 min and 30 cycles of 94°C for 30 sec, 65°C for 30 sec and 72°C for 25 sec. The sequences of the PCR primers used were as follows: forward 5′-CTT CGT GAA CAT TAA CGA CAG GGC C-3′; reverse, 5′-AAT GGC CAC CCT GAC TAA GGA GTG G-3′.

### Statistical analysis

Differences were analyzed with the χ^2^ test for incidence data and the Student’s t-test for comparisons of the means. P<0.05 was considered to indicate statistically significant differences.

## Results

### CDDP-induced activation of AKT in HCT-116 cells

**Figure 1:**
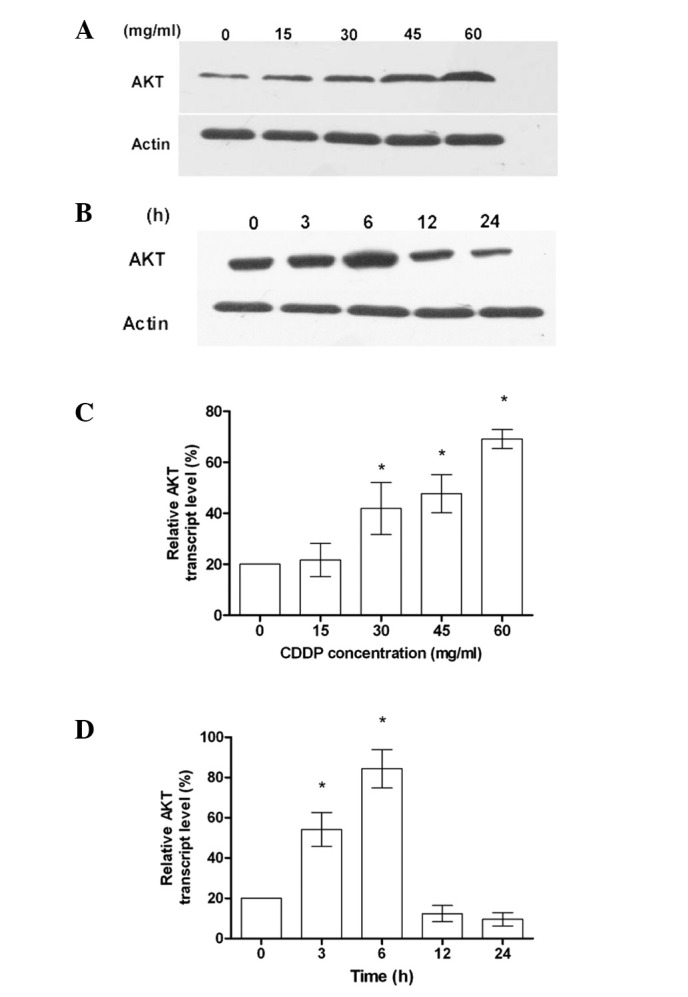
(A and B) Detection of AKT protein expression levels in colon cancer cells (HCT-116) by immunoblotting, with actin used to show equal loading. (C and D) Detection of AKT mRNA expression levels in HCT-116 cells by RT-PCR, with actin used to show equal loading. CDDP, cisplatin.

**Figure 2:**
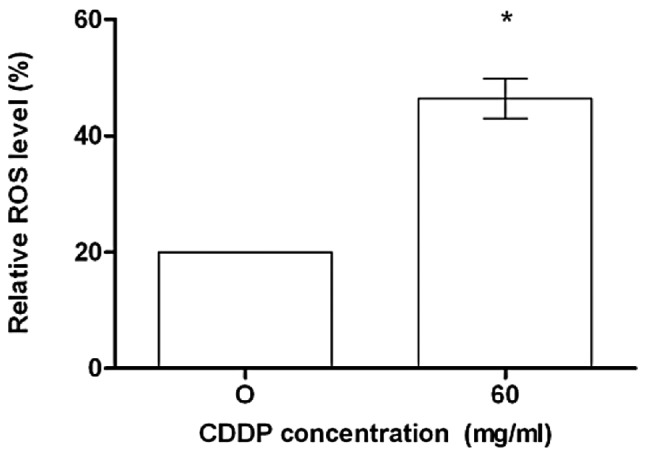
CDDP induces ROS production. HCT-116 cells were treated with CDDP and the production of ROS was measured by DCFDA. CDDP, cisplatin; ROS, reactive oxygen species; DCFDA, dichlorodihydrofluorescein diacetate.

DNA-damaging reagents, such as CDDP, are known to trigger cancer cell death. However, the efficacy of these agents is limited due to chemoresistance, which is associated with AKT overexpression. As shown in Fig. 1A and C, the increased expression of AKT was dependent on the concentration of CDDP. When the cells were treated with CDDP at a constant concentration, the level of AKT expression at 6 h was significantly increased (Fig. 1B and D). These results suggest that AKT gene overexpression is a mechanism for cells exhibiting chemotherapy drug resistance in human colon cancer. Treatment of HCT-116 cells with CDDP induced the generation of hydrogen peroxide and superoxide anions, as measured by the oxidation of the DCFDA. As shown in Fig. 2, constitutive ROS were observed in CDDP-treated cells.

**Figure 3:**
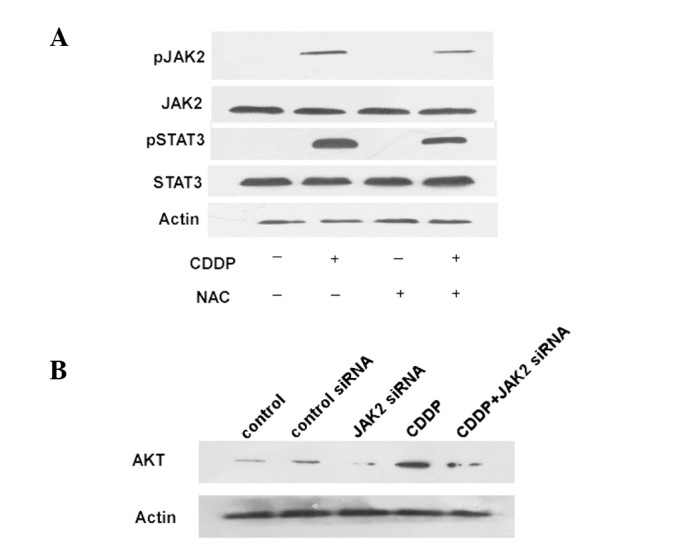
CDDP causes rapid tyrosine phosphorylation of JAK2 and STAT3. (A) HCT-116 cells were treated with 60 mg/ml CDDP and 33 mg/ml NAC. After incubation for 6 h, western blotting probed with phosphotyrosine-specific anti-JAK2 and phosphotyrosine-specific anti-STAT3 antibodies was performed. (B) HCT-116 cells were transfected with JAK2 siRNA and control siRNA and then treated with 60 mg/ml CDDP for 48 h. Western blotting probed with AKT antibody was performed. CDDP, cisplatin; NAC, *N*-acetylcysteine; JAK2, Janus protein tyrosine kinase 2; STAT3, signal transducer and activator of transcription 3; siRNA, small interfering RNA.

To test the hypothesis that the JAK/STAT signaling pathway activation is associated with ROS in HCT-116 cells, growth-arrested HCT-116 cells were treated with 60 mg/ml CDDP or 33 mg/ml NAC at concentrations that were able to significantly affect AKT activation. CDDP caused rapid tyrosine phosphorylation of JAK2 (Fig. 3A). As shown in Fig. 3A, constitutive tyrosine phosphorylation of STAT3 was observed in the CDDP-treated cells. Reducing the expression of JAK2 in colon cancer cells using JAK2 siRNA decreases the AKT expression levels (Fig. 3B). These experiments demonstrate the phosphorylation and nuclear translocation of STAT3 in CDDP-stimulated HCT-116 cells. Taken together with the results shown in Fig. 3, these findings indicate that CDDP activates the JAK2/STAT3 pathway through the generation of ROS in HCT-116 cells.

### STAT3 increases AKT expression

**Figure 4:**
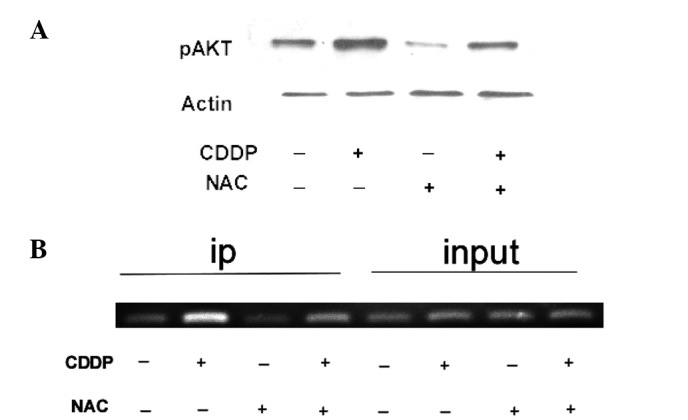
(A) HCT-116 cells were treated with 60 mg/ml CDDP and 33 mg/ml NAC. After incubation for 6 h, western blotting probed with AKT antibody was performed. (B) HCT-116 cells were treated with either CDDP 60 mg/ml or NAC 33 mg/ml. After incubation for 6 h, the cells were subjected to ChIP analyses. CDDP, cisplatin; NAC, *N*-acetylcysteine. Input, non-immunoprecipitated; Ip, immunoprecipitated.

To analyze the transcriptional regulation of the AKT gene, a sequence from the human AKT promoter gene was selected. To demonstrate whether STAT3 binds directly to the STAT3-binding site within the AKT promoter and that AKT is transcriptionally regulated by STAT3, HCT-116 cells were treated with 60 mg/ml CDDP and 33 mg/ml NAC and the ChIP assay, which detects specific genomic DNA sequences that are associated with particular transcription factors in intact cells, was performed. HCT-116 cells were treated with 60 mg/ml of CDDP and 33 mg/ml NAC and immunoprecipitated with a STAT3 antibody. As shown in Fig. 4B, the STAT3 bound chromatin was subjected to PCR using oligonucleotide primers to amplify the region of the STAT3-binding site within the AKT promoter. As shown in Fig. 4A, NAC and to CDDP exposure were compared. In this experiment, CDDP treatment reduced the AKT expression levels compared with cultures exposed to NAC. These results indicate that STAT3 is able to bind to the AKT promoter and CDDP increases the AKT activation.

## Discussion

Reducing chemotherapy drug resistance is a huge clinical challenge. It is well established that chemotherapy induces drug resistance in tumor cells, resulting in treatment failure. In addition, hyperactivation of AKT has been detected when cancer cells acquire chemoresistance (4,7). The present study showed that CDDP stimulates the JAK2/STAT3 pathway, which participates in inducing AKT gene expression. Previous studies (4,16) have shown that CDDP resistance is associated with AKT overexpression. The activation of AKT promotes the development of resistance to chemotherapy treatment (17–19). In the present study, JAK2, activated by CDDP-induced ROS, was associated with STAT3 phosphorylation and the transactivation of a STAT-targeted AKT gene promoter. Reducing the expression of JAK2 using siRNA reduced the AKT expression. These data suggest that the JAK2/STAT3 pathway is a potential target for overcoming chemoresistance.

CDDP stimulates AKT activity in HCT-116 cells, with the peak activity occurring with 200 *μ*mol/l at 6 h. Inhibition of ROS activity by NAC treatment partially inhibits CDDP-stimulated JAK2 and STAT3 activities (Fig. 3A). Based on these findings, it is proposed that ROS may have effects upstream of the JAK2/STAT3 pathway. Alterations to AKT at the protein level have been reported in certain gastric cancers (20). However, small number of tumors have exhibited elevated AKT mRNA levels, which indicates that AKT is regulated at the transcriptional level. Translational regulation of AKT has been well documented in a number of studies (21–23). In the present study, a sequence was selected from the human AKT promoter and a STAT3 binding site was revealed within the promoter. It was notable that STAT3 was not able to bind to the AKT promoter, as revealed by the ChIP assay. The promoter of AKT activity was significantly upregulated by CDDP. When ROS activity was inhibited, the expression of AKT decreased in the HCT-116 cells. The AKT and JAK2/STAT3 pathways are important in cellular processes associated with chemoresistance (24,25). The results showed that the JAK2/STAT3 pathway is able to mediate AKT expression and represents a novel target for overcoming CDDP resistance in human tumors.

The present results indicate that the AKT promoter construct containing the functional STAT3-binding site was activated by CDDP-induced ROS, which are blocked by treatment with NAC. JAK2-mediated phosphorylation of STAT3 is therefore required for AKT promoter activation by ROS. Significant AKT activation was observed in HCT-116 cells treated with CDDP. The observation that AKT expression is enhanced by the JAK2/STAT3 pathway in HCT-116 cells treated with CDDP suggests that the JAK2/STAT3 pathway is a potential target for overcoming chemoresistance.

In conclusion, the present study demonstrated that STAT3 transcriptionally regulates the AKT gene. Blocking ROS with NAC decreases AKT expression. These findings are important for two reasons. Firstly, they provide a mechanistic understanding of the upregulation of AKT. Secondly, the JAK2/STAT3 pathway was also shown to mediate AKT expression, representing a potential target for overcoming CDDP resistance in human tumors.
